# Automated opioid risk scores: a case for machine learning-induced epistemic injustice in healthcare

**DOI:** 10.1007/s10676-023-09676-z

**Published:** 2023-01-23

**Authors:** Giorgia Pozzi

**Affiliations:** Faculty of Technology, Policy and Management, Delft University of Technology, Jaffalaan 5, 2628 BX Delft, The Netherlands

**Keywords:** Epistemology and ethics of ML, PDMP, Opioid risk score, Medical ML, Epistemic injustice, Hermeneutical injustice, Automated hermeneutical appropriation

## Abstract

Artificial intelligence-based (AI) technologies such as machine learning (ML) systems are playing an increasingly relevant role in medicine and healthcare, bringing about novel ethical and epistemological issues that need to be timely addressed. Even though ethical questions connected to epistemic concerns have been at the center of the debate, it is going unnoticed how epistemic forms of injustice can be ML-induced, specifically in healthcare. I analyze the shortcomings of an ML system currently deployed in the USA to predict patients’ likelihood of opioid addiction and misuse (PDMP algorithmic platforms). Drawing on this analysis, I aim to show that the wrong inflicted on epistemic agents involved in and affected by these systems’ decision-making processes can be captured through the lenses of Miranda Fricker’s account of *hermeneutical injustice*. I further argue that ML-induced hermeneutical injustice is particularly harmful due to what I define as an *automated hermeneutical appropriation* from the side of the ML system. The latter occurs if the ML system establishes meanings and shared hermeneutical resources without allowing for human oversight, impairing understanding and communication practices among stakeholders involved in medical decision-making. Furthermore and very much crucially, an automated hermeneutical appropriation can be recognized if physicians are strongly limited in their possibilities to safeguard patients from ML-induced hermeneutical injustice. Overall, my paper should expand the analysis of ethical issues raised by ML systems that are to be considered epistemic in nature, thus contributing to bridging the gap between these two dimensions in the ongoing debate.

## Introduction

The implementation of AI-based methodologies—particularly machine learning (ML) techniques—in healthcare has the potential to provide improved diagnostic accuracy that could by far exceed physicians’ expertise. Particularly impressive results have been achieved, for example, in the field of radiology, pathology, and ophthalmology (Bejnordi et al., 2017; Golden, 2017; Rampasek & Goldenberg, 2018; Singh et al., 2018). According to the current stand, machine vision can interpret specific medical images as accurately as—or even more accurately—than humans (Topol, 2019, p. 47).

Moreover, AI systems implemented in healthcare play a considerable role in managing the challenges raised by the current COVID-19 pandemic (Lim et al., 2022). For example, the MIT Technology Review reports of an AI device used in hospitals in the UK to perform initial readings of a patient’s chest X-rays to be able to recognize the features of COVID-induced pneumonia in the fastest way. As such, delicate and decisive decisions regarding patient triage have been happening according to the recommendation of AI systems, contributing to managing the vast patient loads during the current pandemic (Hao, 2020).

These considerations suggest that we have a *prima facie* moral reason to make use of these systems since they are supposed to provide healthcare professionals with the most suitable and advanced techniques to improve healthcare provision in fundamental medical practices such as diagnostic, treatment recommendations, and prognosis, among others.^1^ However, epistemic limitations connected to how these systems operate are a reason of great concern in the current scientific debate, particularly when assessing the results produced by algorithmic systems implemented in critical areas such as healthcare. In fact, the decision-making logic of ML systems is often epistemically inaccessible to the human knower, constituting the widely discussed problem of epistemic opacity. Indeed, said opacity leads to the concern that high-impact procedures are shifting away from human control. Throughout this article, I take the notion of epistemic opacity as defined by Humphreys (2009) and formally advanced by Durán and Formanek (2018). Drawing on Humphrey’s formulation of the problem (Humphreys, 2004, 2009), these authors particularly focus on the justificatory aspects of epistemic opacity, defining it in terms of accessibility and surveilability conditions on justification. According to their definition of epistemic opacity, a human agent, due to her limited cognitive resources, not only fails to access every relevant step in the justificatory chain but, should accessibility ever be possible, she would not be able to check every relevant passage (Durán & Formanek, 2018, p. 650).

Most machine learning and deep learning algorithms implemented in healthcare are epistemically opaque.^2^ The peculiarity of these algorithms is their ability to change their decision-making rules autonomously as more information is introduced into the system (Alpaydin, 2014). Therefore, these systems can reach a level of complexity that, combined with the processing of huge amounts of data, is not graspable by human cognitive abilities. So understood, the problem of epistemic opacity translates into ethically relevant questions that concern, among others, the degree of trust we are justified to attribute to the outputs produced by these systems and under which circumstances we are epistemically and morally justified in acting upon them (Mittelstadt et al., 2016). For these reasons, debates about the epistemic opacity of ML systems as well as their implementation in highly sensitive fields such as healthcare have, legitimately, gained increasing attention in the academic debate in recent years [e.g., Esteva et al. (2019), Grote and Berens (2020), and London (2019)]. Nevertheless, the question of how genuinely epistemic forms of injustice emerge *due to* the role played by ML systems has not acquired a central stage in the current debate yet.^3^ This paper aims to address the issue of ML-induced epistemic injustice, specifically related to the use of ML systems in the context of medicine and healthcare.

Very broadly, epistemic injustice is lurking and needs to be explicitly addressed as soon as epistemic limitations of ML systems represent an obstacle to the meaningful participation of relevant stakeholders (e.g., physicians and patients) in medical decision-making processes. This happens, I submit, if the system is incontestable from the side of human experts and if it establishes knowledge-creating processes^4^ that systematically exclude patients’ lived experiences as  legitimated sources of knowledge that are qualified to inform decision-making. I consider the conceptual framework of *epistemic injustice* as developed by Miranda Fricker in the field of social epistemology (Fricker, 2007) and its application in the context of ML implementations in healthcare. I am convinced that Fricker’s pioneering work can be pivotal in unveiling subtle forms of injustice that are potentially going unnoticed in the current debate revolving around the epistemology and ethics of ML in the field of healthcare (“Epistemic injustice” section).

Against this background, the main goal of this paper is to address issues of hermeneutical injustice, i.e., a form of epistemic injustice, starting from the consideration of an ML-based tool currently deployed in the USA to predict patients’ risk of opioid misuse, i.e., Prediction Drug Monitoring Programs (PDMPs, see “Injustice in the production of knowledge: the case of PDMPs” section). Through careful consideration of the current flaws identified in this system's functioning, I show that it fulfills three fundamental conditions to identify forms of hermeneutical injustice suffered by patients affected by PDMPs' decision-making (“Defining epistemic injustice in ML” section). I further argue that the hermeneutical injustice these systems bring about is mainly due to the fact that an *automated hermeneutical appropriation* (“Automated hermeneutical appropriation” section) has occurred.

The overall aim of this paper is to show that ML-induced epistemic injustice is a present and pressing concern. In doing so, I also aim to enrich the current landscape of ethical and epistemological issues connected to opaque ML systems implemented in healthcare. This should hopefully motivate further research aiming at providing adequate answers to the concerns raised.

## Epistemic injustice

Drawing on Fricker’s groundbreaking book (ibid.), a large body of literature has arisen, seeking to expand her theoretical framework and trying to find new critical areas in which the analysis of this concept can be helpful to uncover dormant practices that undermine epistemic subjects as knowers. Kidd et al. (2017) point out that “[i]n the era of information and communication, issues of misinformation and miscommunication are more pressing than ever. Who has voice and who doesn’t? Are voices interacting with equal agency and power? In whose terms are they communicating? Who is being understood and who isn’t (and at what cost)? Who is being believed? And who is even being acknowledged and engaged with?” (ibid., p. 1). These questions assume particular relevance if we consider that ML systems are powerful epistemic entities increasingly involved in decision-making processes that strongly impact the life of knowing subjects in a morally salient sense. For these reasons, the phenomenon of epistemic injustice (and the relevant questions it brings about) in the field of ML deserves particular attention.

Fricker (2007) distinguishes two forms of epistemic injustice: *testimonial* and *hermeneutical injustice*. Even though the analysis of ML-induced testimonial injustice is surely worth pursuing,^5^ this paper’s focus is on hermeneutical injustice. Fricker conceptualizes hermeneutical injustice as stemming “from a gap in collective hermeneutical resources—a gap, that is, in our shared tools of social interpretation” (ibid., p. 147). Hermeneutical resources can be defined as cognitive and linguistic resources, i.e., concepts and words we deploy to understand and communicate about the world. They are collectively shared to the extent that they are widely comprehensible to society at large (Mason, 2021, p. 248).

Hermeneutical injustice emerges in two problematic cases that concern either the absence of or a failure to apply shared hermeneutical resources. First of all, issues in terms of hermeneutical injustice arise when a gap exists in the shared pool of said resources. That is to say, subject *S* is experiencing something that she cannot make sense of and, consequently, cannot express to others because what she is experiencing is not part of the shared meanings created and accepted by society. For example, consider the time before postpartum depression (PPD) was recognized as a medical condition (Fricker, 2007, p. 149). At that time, neither the word *PPD* nor the concept of **PPD** was available. Hence, a gap was present in the pool of shared hermeneutical resources leading to a lack of understanding that can amount to a proper injustice. In fact, this disorder was not recognized for a long time as a medical condition requiring due medical consideration because of women’s hermeneutical marginalization in biomedical research. Healthcare professionals did not sufficiently understand the symptoms, and, as a consequence, women clinically diagnosed with PPD often felt ashamed because of the feeling of not being able to meet the standards for motherhood imposed by society. This was due to a lack of conceptual and linguistic tools needed to comprehend one’s own experience and render it intelligible to others (Chung, 2021). In such cases, the injustice amounts to the fact that *S* is taken to be disappointing duties connected to motherhood instead of being recognized as experiencing a particular psycho-physical medical condition for which she cannot be considered morally blameworthy. The impossibility of “giving a name” to this experience obscures understanding and hinders communication practices, leaving women alone with what they are experiencing.

Second, hermeneutical injustice can emerge in different terms, i.e., in the case that there are hermeneutical resources available and widely accepted within a society to conceptually grasp and linguistically express a certain experience *e*. However, subject *S* does not feel represented by these. For instance, this would be the case if her lived experience falls outside the scope of or is not aligned with the socially accepted definition of *e*. Fricker shows this by referring to the definition of homosexuality imposed by society in the 1950s and the protagonist’s experience in Edward White’s autobiographical novel “A Boy’s Own Story” (Fricker, 2007, pp. 163–168). Here, the injustice amounts to the fact that the definition imposed on *S* shadows his own identity and self-understanding. In this case, hermeneutical injustice is not due to a lacuna in collective hermeneutical resources but rather emerges from a failure to apply to one’s own lived experience the concept as it is rendered collectively available (Mason, 2021).^6^

In the face of these considerations, it can be more generally said that in cases of hermeneutical injustice, the subject cannot properly comprehend her own experience and, consequently, cannot render it communicatively intelligible to others. What is important to highlight in this respect is that no agent perpetrates hermeneutical injustice—it is a purely structural notion, according to Fricker. For this reason, in the absence of a clearly identifiable perpetrator, this form of epistemic injustice is particularly difficult to overcome.

The analysis of aspects related to the health condition of patients that put them in a position of epistemic vulnerability that can compromise their epistemic confidence has attracted much attention in the literature (Kidd et al., 2017; Kidd & Carel, 2017; Chung, 2021). That is to say, standard, non-ML-mediated epistemic practices between patients and physicians are already prone to put the patient in a position of epistemic weakness (Carel & Kidd, 2014; Carel et al., 2017).

Against the theoretical background provided by Fricker, I aim to analyze the role that ML systems play as a further authoritative epistemic entity in medical decision-making. In fact, when an ML system offering recommendations and diagnoses^7^ enters this fragile ground, it is paramount to ensure that this does not further weaken patients’ epistemic position. More concretely, should this be the case, it would mean, in its broadest sense, that patients are being attributed a deflated level of credibility. Moreover, it would imply that they are excluded from shared decision-making and are limited in fundamental cognitive activities such as understanding *due to* the role played by the ML system involved in the decision-making process. Considering that ML systems are epistemically authoritative, hardly contestable, and a constitutive part of decision-making procedures that directly impact patients’ lives, we need to ensure that they do not undermine them in their capacities as knowers.

To avoid abstract considerations, the theoretical framework underpinning the identification of forms of epistemic injustice needs to be applied to the careful analysis of concrete cases. Hence, in the following sections, I analyze instances of hermeneutical injustice arising in connection with how ML-based Prescription Drug Monitoring Programs (PDMPs) are currently deployed throughout the US to determine opioid users’ likelihood of overdose and drug misuse.

## Injustice in the production of knowledge: the case of PDMPs

To face the challenges raised by the ongoing opioid crisis pervading the USA [cf., e.g., Vadivelu et al. (2018)], municipalities throughout the country have adopted automated PDMPs to control opioid prescriptions (Oliva, 2022). PDMPs have already been introduced as electronic databases in the 1990s to monitor the prescription of controlled substances to sink the risk of misuse, addiction, and overdose (Haines et al., 2022). The rash advancement of AI and ML in the last decade has led to the implementation of advanced algorithm-based PDMPs (Oliva, 2022). The main goal of these systems is to contribute to containing the opioid epidemic by attributing to patients a risk score to determine their likelihood of developing opioid misuse. The score provided to each patient is supposed to inform medical decision-making regarding which therapies to subject patients to, which medicines to prescribe, and whether a pharmacist should grant patients access to opioids (ibid.).

The dominant algorithmic platform used to determine patients’ risk scores is called NarxCare and is produced by the company Appriss (recently renamed Bamboo Health) (Szalavitz, 2021). The fact that a proprietary algorithm is deployed to determine patients’ likelihood of drug misuse constitutes a black-box since information regarding the data sources used to produce the results is not made publicly available. As such, the latter cannot be reproduced or externally validated (ibid.). Therefore, the users of these systems are not provided with any explanation regarding how the system came to generate a particular output in classifying an individual as having, say, a high risk of drug abuse. Particularly worrisome is also the fact that the proxies adopted to determine risk scores are questionable in themselves (Oliva, 2022). In fact, to estimate patients’ risk scores, factors such as the following are considered: the distance a patient travels to reach a physician/pharmacy, the number of specialists consulted within a specific time frame, payment method used to purchase medicines, number of prescribers, whether the patient in question has a history of sexual abuse or other similar traumatizing events, criminal history, among others (ibid., p. 97). These risk indicators augment stigmatization and discrimination of minorities, disadvantaged socio-economic groups, and patients with a complex medical history, not to mention that they can produce genuinely misleading results. For example, someone living in a rural area is more likely to travel a long distance to purchase her medication. Furthermore, patients with particularly serious pathologies are more likely to consult more than one medical professional to receive the suitable amount of care that their particular condition requires. These, taken for themselves, innocuous facts automatically raise a patient’s risk score because traveling a long distance to purchase medicament and having multiple doctors are indicators of so-called “doctor shopping” behavior, which is taken to be strongly connected to a high risk of drug misuse (Oliva, 2022, p. 97). Other plausible reasons why a patient needs to travel a long distance to receive medical assistance are not considered in the risk score estimation. For these reasons, deploying these risk-scoring systems as a basis to inform medical decision-making can have dramatic consequences for patients, particularly those belonging to minorities and/or socially disadvantaged groups.

As I further consider below, even though Bamboo Health states that “NarxCare scores and reports are intended to aid, not replace, medical decision making” and continues affirming that “[n]one of the information presented should be used as sole justification for providing or refusing to provide medications” (see https://bamboohealth.com/narxcare-and-patients/), the reality of daily medical practice strongly contradicts these claims. In fact, even if Bamboo Health insists that these tools are not conceived of as substituting medical decisions, and the last decision is always in the hands of experienced professionals, physicians are expected to use these systems and consider their outputs. Healthcare providers can prescribe opioids to red-flagged patients at their own peril. The risk of being labeled an overprescriber could have extreme consequences for them and even lead to losing their practicing license (Oliva, 2022, p. 103). As a matter of fact, a study conducted by Picco et al. (2021, p. 8) reports that the number of patients being refused medical assistance and medication supply and of patients being discharged from practice has considerably increased due to the role taken up by PDMP platforms.

Sadly in line with what has been said so far, Szalavitz (2021) reports the story of Kathryn, a young woman suffering from endometriosis, a pathology that causes her severe abdominal pain that she could mostly get under control by being administered oral opioids. On one occasion, when she went to the hospital with severe pain, she was administered opioids to alleviate it. However, a couple of days later, she was dismissed from the hospital with no explanation and still in a precarious health condition. It turns out that, to her unknown, her PDMP risk score resulted in being very high, and on this basis, her gynecologist abandoned her interrupting their relationship (ibid.). Her situation is deeply problematic for multiple reasons. First, she was not informed that an automated system was involved in such a delicate decision-making process in the first place (ibid.), and when she was dismissed from the hospital, she lacked any understanding of what was happening to her. Moreover, it is unclear from which sources NarxCare gathers data to develop risk assessment scores, raising important concerns regarding patients’ right to privacy and informed consent. Even though these are all ethically problematic aspects that require due attention, the focus of my analysis will be limited to whether patients experiencing situations similar to Kathryn’s can be considered a victim of hermeneutical injustice and, if so, how the particular forms of ML-induced epistemic injustice they are suffering can be conceptualized. Frame the issue in question and spelling out its problematic characteristics is the first step to raising awareness of the problem and starting to work toward possible solutions.

## Defining hermeneutical injustice in ML

Let me continue with analyzing the epistemic position of a patient whose risk score has been defined by the PDMP algorithmic system. For the sake of my argument, I consider the situation of a patient that has been, like Kathryn, mistakenly flagged as being at high risk of opioid misuse.

To substantiate the claim that the epistemic authority assumed by opaque ML systems such as PDMPs in decision-making processes in healthcare can lead to a form of hermeneutical injustice at patients’ expenses requires showing that:^8^

1. the ML system involved in a decision-making process (diagnoses, treatment recommendation, among others) holds an *unwarranted epistemic privilege* (it is an epistemic privilege because it can establish meanings and plays a decisive role in shaping shared hermeneutical resources. It is unwarranted because it eludes human intervention);
2. this unwarranted epistemic privilege and the way in which hermeneutical resources are established hinder *understanding* and *communication* among relevant stakeholders involved in decision-making processes (physicians, patients). This renders significant aspects of social experience not intelligible to them.^9^ If this is the case, the combination of these factors points to the fact that an *automated hermeneutical appropriation*^10^ has occurred;
3. the interplay of 1. and 2. leads to considerable disadvantages, particularly at the expense of hermeneutically marginalized groups (e.g., patients, minorities, already stigmatized groups due to substance use disorders, etc.).

I argue that these conditions are all met in the case considered, and we are therefore dealing with a clear instance of hermeneutical injustice. I address these points in turn.

### Condition 1: PDMPs and unwarranted epistemic privilege

Let me start with condition 1. In order to be able to assess whether the ML system in question holds an *unwarranted* epistemic privilege, it is essential to have a clearer view of when such a privilege can be considered as being indeed unwarranted and when not. For example, it is highly plausible to take that a physician holds a *warranted* epistemic privilege when interpreting, say, the meaning of a patient’s CT scan and acting upon said interpretation (Carel & Kidd, 2014, p. 536). In this case, the physician’s epistemic privilege is justified in her capacities and expertise that render her epistemically well-positioned in offering a grounded interpretation of a CT scan. That is to say, her epistemic privilege is warranted by a combination of long medical training, experience with different forms of patients’ diseases, the information provided by scientific literature, and similar other sources, which contribute to rendering her an overall reliable and trustworthy professional. On the other hand, an example of an unwarranted epistemic privilege could be the role that standardized protocols play in certain healthcare practices. One could argue that standardized protocols constrain patients’ testimony of lived experiences in rigid schemes that cannot account for their more subjective experiences (Moes et al., 2020). If, due to the rigidity of protocols, the subjective experience of patients is not considered a legitimated source of knowledge (because of, for example, the difficulty or impossibility of expressing it in quantifiable terms) and, as such, is *excluded altogether* from informing decision-making, one can conclude that standardization holds at least the possibility of taking up an unwarranted epistemic privilege.^11^ More precisely, this would be the case if standardization had a considerable bearing on which forms of knowledge are recognized as such and can inform decision-making and in the case that the form in which the evidence is presented is decisive in respect to epistemic participation in medical decision-making.

An unwarranted epistemic privilege can come to light also in connection to how hermeneutical resources, i.e., concepts and meaning, are established and shared. As such, considerations regarding the existence of an unwarranted epistemic privilege seem to apply to how the PDMP system under scrutiny establishes meanings connected to a patient’s drug misuse. In the following, I argue that these systems enforce their meaning of what opioid addiction amounts to on both patients and physicians, exceeding the decision power of a system that should be functional to improving and supporting human decision-making and not replacing or impairing it, paving the way to cases of hermeneutical injustice.

In the case considered, a problem connected to hermeneutical resources is not to be traced back to a lack thereof. In fact, we certainly have appropriate linguistic and conceptual tools, such as, for instance, the word *addiction* that is suitable to articulate the concept of **addiction**. We also know that the latter amounts to clearly definable criteria above and beyond the fact that the concept has a grounded medical definition (I take addiction, but it could also be the words and concept related to what is medically understood under substance use disorder (SUD), for example). The problem amounts to the fact that the ML system defines the concept of **addiction** according to not shared metrics so that the *meaning* attributed by the system to a red flag can considerably shift away from the widely shared, accepted, and medically grounded meaning of the same concept, without the possibility for stakeholders affected to amend this shift.

This comes to light in the face of several considerations. First of all, in defining parameters for determining, for example, the threshold that allows the system to differentiate between concerning and not concerning cases in connection with the risk of opioid misuse, value-laden choices are necessarily made. These range from the choice of the proxies used to determine the risk scores (along with each proxy’s weight in determining the final score) to the model’s design and the definition of the goal that the system should fulfill. How this is done by developers of the system, the company owning it, or the system itself due to its self-learning and adaptive mechanisms has the effect that this is not rendered explicit and transparent to the stakeholders directly involved in and affected by these systems’ decision-making. It follows that this entails failing to ensure that the system’s representations indeed mirror the accepted definition of the concept.

Furthermore, Oliva (2022) points out that the end goal of these systems is to reduce opioid prescriptions, regardless of the consequences that this has for the patients affected by the risk scores produced. This means that as long as it can be shown that due to the use of PDMPs, physicians issue fewer opioid prescriptions, the system is deemed *efficient*. However, this is also a value-based choice that does not show that patients flagged as being at a high risk of opioid misuse indeed are running this risk (according to the collectively shared meaning of concepts such as **addiction** or **SUD**). NarxCare does not assess whether clinical prescription decisions improve or exacerbate patients’ pain, mental health, or overall quality of life (ibid., p. 88). That is to say, the measured effectiveness of the system is limited to the number of prescriptions issued, without further investigating whether a particular patient actually benefits from the interruption of opioid medicament and she was indeed at risk of opioid misuse, or she was red-flagged due to correlations established by the system that are not indicative of opioid addiction or misuse. It follows a shift in the meaning of addiction or SUD as it is established by the system toward its end goal of reducing prescriptions. However, patients are confronted with a risk score that is treated as grounded knowledge that reliably mirrors their drug consumption levels.

Consequently, a misalignment can emerge between the knowledge that a patient (like Kathryn) has about herself and a risk score taken to indicate drug misuse and addiction, according to the definition established by the PDMP system. It follows that ambiguous and heterogeneous information that can be tangential and not representative of establishing a risk assessment is treated and elaborated as a form of *knowledge* suitable to guide medical decisions. This is, to my mind, the extent to which algorithmic PDMPs such as NarxCare hold an unwarranted epistemic privilege.

The latter is considerably reinforced by the fact that PDMP predictive platforms are “the only law enforcement-developed digital surveillance systems that health care providers have ever utilized to diagnose and treat patients” (ibid., p. 51). Thus, even though, in theory, they should serve as tools to support physicians’ decision-making, in practice, they strongly limit patients’ and physicians’ possibilities to critically question the risk score they assign. Bottom line, they take up a decision power that sidelines the weight that patients’ knowledge—in the form of their testimony, personal (moral and epistemic) values, and lived experiences—can have in the process of medical decision-making.

Furthermore, the concern emerges that how the ML systems under scrutiny produce what is considered legitimate knowledge able to inform medical decision-making is unidirectional: physicians are provided with a score that cannot be revised according to relevant information that could potentially overturn it. That is to say, users are not able to feed back into the system valuable information that should be taken into consideration, making the user just a recipient of knowledge coming from unknown sources, but they do not get to actively influence the knowledge-producing process itself (Pozzi & Durán, under review).

In line with these considerations, it can be stated that the ML system involved in the case under consideration holds a clear unwarranted epistemic privilege: it establishes what it means to be at a high risk of drug misuse by attributing to each patient a risk score related to their likelihood of misusing opioids based on questionable proxies and without allowing for contestability of the results. The fact that the consideration of these systems’ outputs is legally enforced on physicians exacerbates the weight of their authority even further.

Condition 1 is thereby fulfilled.

### Condition 2: Understanding and communication impairments

Let us move forward with the consideration of condition 2. As already mentioned, being NarxCare a proprietary algorithm, the possibilities to get meaningful insight into how it works and which criteria were decisive in estimating a patient’s high-risk score are very much constrained. That is to say, in this case, technical opacity—due to the black-box nature of ML algorithms used in generating the risk scores—is reinforced by the unwillingness of the company that owns these systems to disclose information regarding their functioning (Oliva, 2022). Regardless of the sources of the opacity of these systems, the result is that users involved in and affected by the system’s decision-making processes lack the explanatory resources needed to have a proper understanding of how its outputs are created and, as such, are not able to assess whether these are justified or not. Thus, these considerations imply that they cannot show that the systems’ results have been produced, in a particular case, by the interplay of factors not indicative of drug misuse. This leaves patients and physicians in the dark regarding what actions can be legitimately taken upon the generated output and how patients can question a risk score they do not identify with.

The general lack of understanding leads to the fact that communication between patients and healthcare providers is strongly constrained. There is relevant literature pointing at communication difficulties emerging from the ubiquitous use of PDMP systems throughout medical practice, which are particularly concerning and indicative of patients’ potential to suffer epistemic injustice. After conducting interviews with medical professionals using PDMPs as a basis for decision-making, Hildebran et al. (2016) individuate detrimental communication styles that are, to my mind, representative of the disruptive role taken up by these systems in mediating interactions between patients and physicians. The quotations below are particularly concerning expressions of how these systems’ mediation in medical practice can be detrimental to a “good” patient-physician relationship.^12^ As pointed out by these authors, they amount to avoidance and a confrontational communication style [cf. also Picco et al. (2021, p. 14)], both of which are particularly concerning and indicative of hermeneutical injustice. In the context of the interviews conducted by Hildebran et al. (2016) and Hildebran et al. (2014), physicians stated that:

It’s a cat and mouse thing. So I keep it [the PDMP] secret as much as possible (Hildebran et al., 2016, p. 2063) (*avoidance*).

I will leave the room to get something and pull it [PDMP report]. I may confront them if it seems like they’re lying and say, “Well here’s what I got here. It seems like you haven’t been very honest with me and so I’m not going to provide you with prescriptions.” (Hildebran et al., 2014, p. 1183) (*confrontational*).

The first communication style, avoidance, completely closes down communication between patient and physician. The patient in question is not informed of the existence of an epistemic authority, i.e., the PDMP producing the scores, that has a strong or even *the* final say on crucial matters regarding their mental and physical well-being. On top of this, the risk score is not critically scrutinized by the physician. The latter could, in fact, gain fruitful information regarding the patient’s condition and drug consumption by engaging in an open discussion with her, showing empathy and understanding for a possible problematic situation in which she is in, being this, arguably, an essential aspect of the patient-physician relationship.^13^ In such cases, the patient is not even given the possibility to share and communicate the knowledge she possesses about her current state, which could show her miscategorization as a red-flagged patient. This kind of communication style shows how the role assumed by PDMPs encourages physicians’ disengagement with their patients, with detrimental consequences for the latter. In fact, it is practically impossible for the patient to make sense of why she is being denied medication or medical assistance altogether, being wholly excluded from the communication practice. It is evident that this can create a gap in understanding and sense-making of one’s own situation.

While avoidance suffocates any possibility for the patient to react to a potentially inaccurate risk score, a confrontational communication style, along the lines of approaches similar to the one mentioned in the passage reported, seems to be strongly conducive to distrust and credibility deficits on the side of the patient (Pozzi, 2023). This is the case even if, at least in principle, she is provided with the possibility to argue against a possible risk score with which she does not identify. In the face of a red flag that categorizes a patient as being at a high risk of drug misuse, it will be particularly challenging for her to argue otherwise in the case that the risk score attributed to her does not depict her actual drug consumption, particularly if the physician attributes *by default* more credibility to the PDMP, as it seems to be the case in the passage previously quoted. In this sense, we could say that PDMPs directly influence the credibility judgments of healthcare professionals and could exacerbate already existing prejudices. Concretely, this means for patients that the humane, empathetic, communicative experience between them and their physicians that is paramount to coping with their mental and physical condition of being ill is strongly impaired.

Drawing on these considerations, the risk of epistemic injustice is evident when a stigmatizing authority (i.e., PDMPs) plays a role in mediating decision-making processes in medical care. In fact, above and beyond possible (subconscious) prejudices of healthcare professionals towards socially disadvantaged groups due to their perceived social identity (Kidd & Carel, 2017), a risk score is attributed to them, and, as previously mentioned, this tends to disadvantage members of said groups. It seems indisputable that the risk score further deflates their credibility and weakens their epistemic position.

These considerations indicate communication difficulties emerging from the role that PDMPs play and show that condition 2 for hermeneutical injustice is also fulfilled.

### Condition 3: Hermeneutical disadvantage

Condition 3 aims at capturing how the interplay between the considerations expressed in the analysis of conditions 1 and 2 constitutes a disadvantage, particularly for members of minorities and social groups vulnerable to discriminatory practices. From what has been previously pointed out, it seems plausible to take that the decision power of these ML systems, combined with their incontestability, provides them with an epistemic authority that imposes a form of unchangeable knowledge. Consequently, patients are excluded from the process of informing medical decision-making and are *hermeneutically marginalized*. Kathryn was hermeneutically marginalized at the moment in which, without receiving any explanation whatsoever, she was sent home from the hospital. Her marginalization continues today since even though she could grasp why the system was erroneously attributing her a high score (she has sick pets that need strong medicament),^14^ she could not find a way to clear her record. Moreover, her knowledge continues to be ignored every time she unsuccessfully seeks the support of a physician ready to prescribe her the medicines she needs to cope with the pain caused by her complex medical condition (Szalavitz, 2021). Kathryn’s situation represents a palpable instance of hermeneutical injustice.

In the face of what has been said so far, the nature of the disadvantage can be captured in epistemic, moral, and practical terms. As already mentioned, an epistemic disadvantage is at play since patients’ knowledge is not considered in informing the PDMP’s risk score. It follows that patients’ testimony, personal values, and lived experiences are not accounted for as a legitimate source of knowledge that is able to contest an unjust risk score.

From a moral point of view, these systems fuel stigmatization, discrimination, and unfair treatment, particularly for already disadvantaged societal groups. For instance, women are particularly exposed to miscategorizations by the PDMP due to the proxies used. The fact that sexual trauma is considered an indicator that raises the likelihood of drug abuse disadvantages women. As a matter of fact, they are, on average, more likely to report sexual abuse and seek psychological support compared to men (Oliva, 2022). This means that they will, by default, have a higher risk score than men, a fact that reinforces existing inequalities, increasing the probability that they are thereby denied medication on illegitimate grounds. Gender-related stereotypes connected to women’s perceived emotional instability or their alleged tendency to’exaggerate pain’ have led to considerable and persisting disparities in pain management already in not ML-mediated processes (Lloyd et al., 2020).

Moreover, it seems clear that the consequences of clinical dependence on models that generate high false positive rates by mischaracterizing patients with, for instance, low-risk complex chronic pain as high-risk opioid use disorder are particularly severe (Oliva, 2022, p. 105). Indeed, this further contributes to stigmatizing and hermeneutically marginalizing already fragile categories of epistemic subjects,^15^ strongly constraining their ability to grasp and make sense of what is happening to them. As such, PDMPs reinforce prejudices and discrimination already rooted in our social practices, leading to their systematization. In fact, since PDMP systems are used by physicians almost on a daily basis and are treated as evidence that directly influences medical decision-making, these kinds of biases are likely to propagate considerably, escaping the critical scrutiny of physicians that are possibly aware of these issues and could actively engage in preventing their occurrence. Of course, the same applies to ethnic inequalities in pain care (Mossey 2011) that see, for example, patients of color face considerable obstacles in receiving access to pain medication and a lower quality of care more generally (Oliva, 2022, pp. 94–95).

Finally, as is evident in Kathryn’s case, patients’ disadvantage can express itself in very practical terms: in the denial of medical delivery, in patients’ abandonment, and in the condemnation to live with unbearable pain that could be otherwise alleviated. This can lead to very damaging consequences, such as increased suffering, the feeling of not being heard and understood, and being the victim of a system in which one does not get to play a role as an active epistemic subject but is rather the object of decisions that affect their lives to a great extent.

The fulfillment of these conditions shows that an ML-induced hermeneutical injustice is in place in the case under scrutiny.^16^

## Automated hermeneutical appropriation

What has been said in the previous sections points to the fact that an automated hermeneutical appropriation occurs in the case considered. Under this umbrella term, I understand the interplay of factors that are directly conducive to hermeneutical injustice, specifically *due to* the ML system’s role in taking over aspects of the patient-physician interaction that should, arguably, remain under human control. 

This relates to what has been said in “Condition 1: PDMPs and unwarranted epistemic privilege” and “Condition 2: Understanding and communication impairments” sections regarding the unwarranted hermeneutical privilege taken up by PDMP systems and the communication difficulties that emerge between patients and healthcare professionals due to the role they play in mediating healthcare encounters. Both the idea of causing a misalignment between collectively shared meanings and the meaning established by the PDMP and constituting an obstacle in the process of understanding, point at a role taken up by the system that, very much intuitively, *exceeds* its allegedly intended purpose of supporting medical decisions.^17^

However, ML-induced epistemic injustice cuts particularly deep because it affects not only members of disadvantaged social categories (such as, for instance, patients with substance use disorder). It also negatively impacts favorably positioned epistemic agents such as physicians that otherwise could, through virtuous behavior (Fricker, 2007, p. 169), mitigate the injustice experienced by patients as vulnerable epistemic subjects. This last point further indicates of an automated hermeneutical appropriation and requires further elucidation.

In an attempt to clarify under which conditions an epistemic injustice can be considered indeed an *injustice* in a proper sense, Byskov (2021) formulates five conditions, one of which is, to my mind, particularly suitable to capture the wrong experienced by physicians as epistemic subjects, i.e., what the author calls the *stakeholder condition*. Byskov defines it as follows: “In order for someone to be unjustifiably discriminated against as a knower, they must be somehow affected by the decisions that they are excluded from influencing” (ibid., p. 8).

Physicians’ stakeholder rights should, intuitively, encompass the fact that they are entitled to actually influence medical decision-making if they are to be considered epistemic and morally responsible for their decisions. In the case discussed, physicians are not excluded from straightforwardly influencing decisions since they have, at least formally, the last word on whether or not a patient will be granted opioid prescriptions. However, they are expected to make decisions regarding a patient’s prescription without being able to do so in a system-independent way.

Kathryn’s doctor does not have meaningful insights into why the PDMP system attributed her a high-risk score for drug misuse. Nevertheless, she is supposed to act upon the outcome produced by the system (to her unknown of the relevant factors that led to it). Even if she makes the final decision regarding Kathryn’s treatment, she does not get to influence the decision-making process itself in an active way since she is compelled to act according to the risk score provided (Szalavitz, 2021). Hence, physicians themselves can also be considered victims of this system since “(d)epending on the State specific legal requirements of the PDMP, the PDMP database may generate an automated alert to notify either health and/or law enforcement agencies of suspicious prescribing.” (Haines et al., 2022, p. 2).

Under these circumstances, it is difficult to see to what extent a physician actively influenced the decision-making process when acting according to the risk score generated by PDMPs. Nevertheless, she will indeed be *directly affected* by the consequences of the decision taken because she is likely to be considered blameworthy if the decision made has negative consequences for the patient. Therefore, due to her stakeholder rights, she is supposed to have the possibility to be involved in the decision-making process in a meaningful, genuinely agential way. This does not seem to be the case due to the incontestability of PDMPs and their law enforcement power. These considerations shed light on a further dimension of the system’s hermeneutical appropriation to the extent that physicians are, at least partially, deprived of their stakeholder rights. The fulfillment of the stakeholder condition points to the fact that also physicians are, to a certain extent, experiencing epistemic injustice.^18^

The experience of seeing their epistemic authority undermined by an inscrutable and incontestable epistemic entity in a systematic way could have disruptive consequences for physicians’ professional identity. Moreover, it also entails the possibility of their deskilling and disengagement, eliciting the tendency to evade responsibilities that would otherwise be a constitutive part of their professional role (it has already been pointed out in the discussion of condition 2 how the role of PDMPs leads to physicians’ disengagement with their patients).^19^

Physicians’ epistemic dependence upon PDMPs has a considerable impact on patients. While ML-based PDMPs deprive patients of the conceptual tools needed to understand why they are red-flagged in the case that the *knowledge* they possess about themselves is not aligned with the score they are stigmatized by, there are no options available to the patient to come out from this circle exactly because, crucially, physicians themselves are epistemically dependent on them. For this reason, their ability to potentially counteract a hermeneutical injustice suffered by their patients is very much constrained.

This indicates that a virtue theoretical approach (Fricker, 2007, p. 174) toward mitigating ML-induced hermeneutical injustice suffered by patients is insufficient. Fricker sees the virtue of hermeneutical justice as fundamental to opposing epistemic injustice. She takes this virtue to be corrective in nature to the extent that a virtuous attitude of a hearer showing awareness of the social situation of a speaker and “a more pro-active and more socially aware kind of listening” is able to partially compensate disadvantages emerging from hermeneutical injustice (Fricker, 2007, p. 174). In healthcare encounters, this would require physicians to be particularly aware of a patient’s possible hermeneutical marginalization and their active effort to show understanding for their situation to overcome it.

However, a solution to the issues pointed out in this paper needs to go beyond the virtue theoretical approach indicated by Fricker. In fact, her approach seems to be limited to cases of epistemic injustice emerging in exclusively human-centric epistemic environments. In the case of interest, the difficulty in identifying the oppressing agent and the impossibility for patients to seek recourse to epistemically authoritative agents such as medical professionals (since they are themselves epistemically dependent on the systems) renders the ML-induced injustice they experience even more wide-ranging and difficult to mitigate.

## Final remarks

The overarching goal of this paper was to shed light on issues understood in terms of hermeneutical injustice and brought about by ML systems implemented in medicine and healthcare. To substantiate my argumentative aims, I analyzed in detail a particularly concerning ML-based system currently deployed throughout the USA to produce patients’ risk scores of opioid addiction and misuse. Since physicians are expected to consider these systems’ outputs to inform their medical decisions, it is paramount to critically scrutinize whether they increase patients’ vulnerability to forms of epistemic injustice.

In order to convincingly argue that this is the case, I showed that three main conditions to recognize instances of hermeneutical injustice are met in the case under scrutiny. PDMPs hold an unwarranted epistemic privilege (condition 1) that impairs understanding and fundamental communication practices among patients and physicians (condition 2) and, finally, constitutes hermeneutical disadvantages, particularly for vulnerable social categories (condition 3).

I further argued that ML-induced hermeneutical injustice is to be directly traced back to an automated hermeneutical appropriation from the side of the system. The latter reveals in the way in which hermeneutical resources are established by the system and how it deprives human agents of understanding and hinders their communication practices. On top of this, it deprives physicians of the possibility to actively safeguard patients that are victims of the injustice the ML system brings about since the former are themselves subordinated to the system’s epistemic authority. Crucially, this strongly limits physicians’ possibility to resist hermeneutical injustice through virtuous behavior, as Fricker conceptualizes in her human-centric approach.

More needs to be said regarding how these issues take shape in epistemic practices that see ML systems as powerful and ubiquitous epistemic entities. However, I hope this paper could show the importance of further pursuing the highlighted issues and encourage further research to work towards technically feasible solutions with the aim of overcoming the difficulties recognized.

## Footnotes

1 So understood, the use of computationally powerful AI systems is very much aligned with the fundamental medical principle of beneficence (Lawrence, 2007). See also Van den Hoven (1998, p. 100).

2 Of course, not all AI implementations are epistemically opaque. Methods like simple naive Bayes classifiers, decision trees, linear regressions, and rule lists are usually interpretable (Lipton, 2018; Páez, 2019). Nevertheless, since most of the AI systems currently deployed in healthcare are either machine learning or deep learning systems, I am interested in the analysis of implementations that are epistemically opaque to human agents.

3 Of course, there are some exceptions. For example, the recently published and insightful paper by Symons and Alvarado (2022) initiates a discussion on epistemic injustice in data science technologies.

4 An ML system’s knowledge-creating process can be, for instance, how a scoring system attributing to people's risk scores of being at drug abuse elaborate information regarding a particular subject leading to a precise output. The piece of knowledge produced by the system would be, in this case, the score attributed to said individual. I extensively discuss this case in the sections below.

5 Fricker defines testimonial injustice as occurring when a speaker is downgraded in her role as a knower due to prejudices a hearer holds related to her social identity (Fricker 2007). Most notably, testimonial injustice emerges when a person is granted less credibility for epistemically invalid reasons such as her race, gender, or social status. In this contribution, I focus exclusively on how hermeneutical injustice can be machine learning-induced in the particular case of interest. However, also a testimonial injustice is in place. I discuss this form of injustice in detail elsewhere (Pozzi, 2023).

6 Fricker does not explicitly recognize hermeneutical injustice as a failure in the application of available conceptual and linguistic resources. However, I follow here Mason (2021)’s interpretation as I think it captures at best this second kind of issue that is not related to a lack of hermeneutical resources but rather to a misalignment between the definition available and the subject’s experience.

7 Here, a clarification is in order. Throughout this paper, with phrasings such as, for instance, “an ML system *provides* recommendations”, I do not mean to explicitly attribute any form of agency to ML systems. The issue of whether or not ML systems as technical artifacts can be attributed some degree of agency is a thorny and highly debated issue, towards which this paper remains neutral. I thank an anonymous reviewer for encouraging me to clarify this point.

8 The following conditions are a revised version of the ones formulated by Wardrope (2015, p. 344) along Fricker’s lines. I revise them to capture the role played by the ML system in question in being conducive to hermeneutical injustice. These three conditions are closely related, and I make a clear distinction between them for analytic purposes.

9 In which ways this happens is, of course, context-dependent. In the following, I discuss how this is happening in the case under scrutiny.

10 I discuss what automated hermeneutical appropriation amounts to in more detail in “Automated hermeneutical appropriation” section.

11 Of course, whether this amounts to an unwarranted epistemic privilege or not in a particular case is context-dependent. It surely strongly depends on how healthcare professionals deal with the information gathered in protocols and with other forms of knowledge that exceed them (say, whether they take the time to engage with the patient in question in order to account for the knowledge they possess and could not be captured through the rigidity of protocols. In the latter case, the potential unwarranted privilege of these would be compensated through appropriate conduct from the side of the physician).

12 In the last decades, a paternalistic approach in medicine has been slowly replaced by a more active role taken up by patients toward shared decision-making. The latter is particularly ethically relevant as it enables patients’ autonomy and self-determination (McDougall, 2019). The communication styles ensuing from the increasing use of PDMPs for medical decision-making seem to shift patient-physician interactions back to more paternalistic models. While I cannot pursue this issue further here, I think it is important to consider it.

13 This seems to be the case if we take a deliberative approach to shared decision-making as the paradigm underpinning the patient-physician relationship (Emanuel & Emanuel, 1992).

14 A brief side note is due here. Not every patient is in the condition of fighting against a risk score that is not perceived as depicting one’s own actual opioid consumption as Kathryn did. This could be the case due to different factors depending on one’s own situation, such as, for instance, physical and mental condition or technical literacy. Therefore, it is paramount to give voice to injustices that can arise from the fact that said systems make it so difficult for stakeholders affected by a wrong risk score to show otherwise.

15 On how the risk of epistemic injustice is particularly present for chronic pain patients see Buchman et al. (2017).

16 Of course, in the case considered, the issues recognized in terms of hermeneutical injustice are not to be restricted to the flaws inherent to the ML system considered in isolation. In fact, these technologies are sociotechnical systems in which technical and institutional design must play a decisive role in mitigating the issues pointed out, along with suitable regulations and correct deployment by relevant institutions (Van de Poel, 2020).

17 See, again, NarxCare’s website: https://bamboohealth.com/narxcare-and-patients/.

18 Of course, the consequences of the injustice experienced by physicians in terms of stakeholder rights limitations are not comparable to the hermeneutical disadvantages that afflict patients since the former are still members of a socially privileged group.

19 While a thorough analysis of these systems’ impact on physicians’ professional role is paramount to capturing the nature of the injustice they experience, I cannot pursue this issue further here.
